# Indomethacin induced ductus arteriosus closure in midgestation fetus

**DOI:** 10.1002/ccr3.1385

**Published:** 2018-01-31

**Authors:** Jeanette I. Beaute, Kevin G. Friedman

**Affiliations:** ^1^ Boston Children's Hospital Boston Massachusetts 02115; ^2^ University of Massachusetts Medical School Worcester Massachusetts; ^3^ Harvard Medical School Boston Massachusetts 02115

**Keywords:** Ductus arteriosus closure, fetal cardiology, fetal procedures, indomethacin, right heart failure

## Abstract

This article reports a rare but potentially serious complication of ductus arteriosus closure resulting from second‐trimester indomethacin exposure. Serial echocardiograms are indicated to monitor for development of right heart dysfunction and to ensure delivery prior to the onset of right heart failure and hydrops fetalis.

## Case Report

A 34‐year‐old, gravid two, para zero woman was referred to our center for fetal echocardiography at 19 weeks due to concern for new right ventricular (RV) dilation and tricuspid regurgitation after recent radiofrequency ablation (RFA) for twin‐reversal arterial perfusion (TRAP) sequence. The pregnancy was a monochorionic diamniotic gestation. At 7‐week gestation, twin B was found to be acardiac and anencephalic consistent with TRAP. Fetal echocardiogram for the pump twin at 17 weeks showed cardiomegaly but a structurally normal heart with normal valvar and ventricular function. RFA of the vascular supply to the acardiac twin was successfully performed at an outside facility without acute complication. Peri‐procedural indomethacin was administered for three days as a tocolytic.

**Figure 1:**
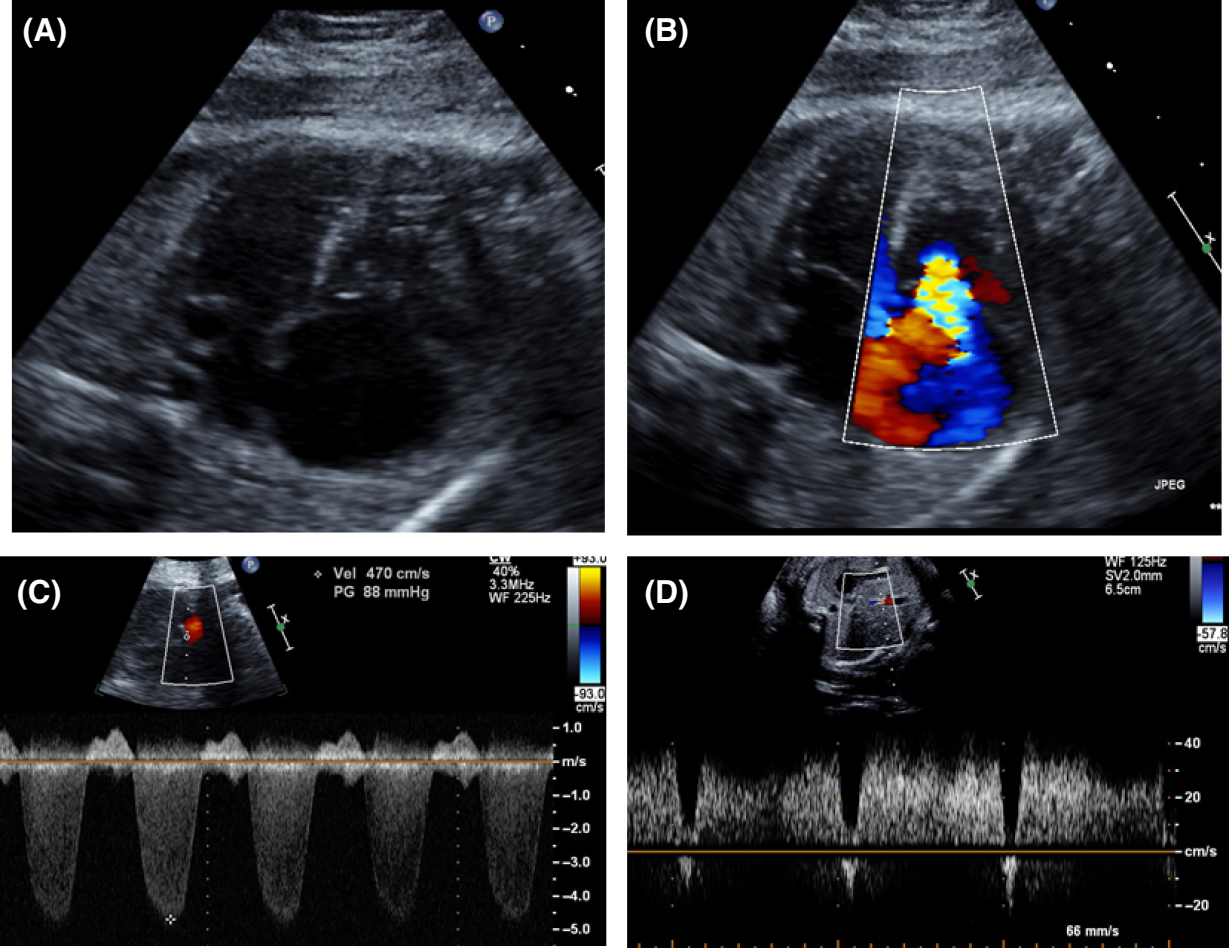
Fetal echocardiogram at 22‐week gestation. (A) Four‐chamber view showing severe right atrial dilation, dilated tricsupid valve, and right ventricular hypertrophy. (B) Four‐chamber view with color flow on the tricuspid valve showing severe tricuspid regurgitation. (C) Continuous‐wave Doppler of the tricuspid regurgitation jet showing severe right ventricular hypertension. Estimated right ventricular pressure is 88 mm Hg plus the right atrial v‐wave. (D) Doppler flow profile of the ductus venosus showing a‐wave flow reversal.

Approximately ten days after the RFA was performed, the patient was referred to our Fetal Care Center for fetal echocardiography due to new RV dilation and tricuspid regurgitation (TR) in the pump twin. Echocardiogram showed moderate TR, RV hypertrophy and hypertension with TR jet velocity indicating RV pressure ~80 mm Hg plus the right atrial v‐wave. Serial echocardiograms over the remainder of gestation showed worsening (severe) tricuspid regurgitation, continued severe RV hypertension as well as progressive RV hypertrophy and dysfunction. No patent ductus arteriosus (PDA) could be identified on the initial or follow‐up echocardiograms. A presumptive diagnosis of premature PDA closure related to peri‐procedural indomethacin was made. At the last fetal echocardiogram performed at 32 weeks, there was severe tricuspid regurgitation, severe RV hypertrophy and hypertension, mild‐to‐moderate RV dysfunction and no pericardial effusion or hydrops (Fig. 1).

The viable twin was delivered at 34 weeks via emergent Cesarean section due to nonreassuring fetal heart rate. Upon delivery, APGAR was eight at one and 5 min. Postnatal echocardiogram shortly after birth showed: no PDA, severely dilated right atrium, severe TR, severe RV hypertrophy and hypertension, and mild‐to‐moderate RV dysfunction. Left heart structures were normal. The neonate was administered 24 h of nitric oxide and remained on supplemental oxygen via nasal cannula. Oxygen saturations were ~80% due to bidirectional patent foramen ovale flow (PFO). With supplemental oxygen over the first seven days of life, oxygen saturations improved to ~93% and there was marked improvement in the degree of RV hypertension. The patient is now 3 years old and has undergone no cardiac procedures. At the most recent follow‐up, growth and development are normal. The oxygen saturation is now 98%. Echocardiogram shows moderate TR with normal predicted RV pressure, borderline dilated RV with normal function and no hypertrophy.

## Discussion

Indomethacin is well established as an effective tocolytic agent 1, 2, 3. Indomethacin has a long track record in prevention of spontaneous preterm labor and more recently has been administered to prevent preterm labor during several types of midgestation fetal interventional procedures. Prior studies have reported that third‐trimester administration of indomethacin can lead to constriction of the ductus arteriosus that is generally reversed by removing the drug 2, 4, 5. Indomethacin sensitivity is associated with gestational age as the incidence of ductal constriction increases with increasing gestational age. The risk of PDA restriction/closure with indomethacin is generally thought to be low in early second trimester 4, 5. Current guidelines from the American College of Obstetricians and Gynecologists recommend an upper gestational age threshold of 32 weeks for tocolytic use of Indocin 6.

In this case, we present an 18‐week fetus who developed irreversible PDA closure due to peri‐procedural indomethacin administration. The fetus developed severe cardiac sequelae in utero due to PDA closure and resultant severe RV pressure load. The sequelae included RV hypertrophy and dysfunction as well as severe TR and right atrial dilation. Fortunately, the fetus did not develop frank RV failure or hydrops fetalis which can occur with severely increased RV afterload.

In utero ~75% of RV output bypasses the high‐resistance pulmonary circulation through the patent ductus arteriosus. With in utero PDA closure, the entire RV output (~60% of combined cardiac output) is forced through the high‐resistance pulmonary circulation. This obligatory increase in pulmonary blood flow combined with the fixed high pulmonary vascular resistance leads to a marked increase in RV afterload. This fetus manifested typical consequences of increased RV afterload including RV hypertrophy and systolic dysfunction, as well as TR, and right atrial dilation due to TR and noncompliance of the hypertrophied RV. In some cases, this cascade of right heart dysfunction can lead to overt RV failure and hydrops fetalis 7, 8. Fortunately, this fetus did not develop hydrops fetalis and was delivered at only mildly preterm.

Following birth, the neonate did not require any cardiac interventions. Normally, pulmonary vascular resistance (PVR) decreases within the first days of life; however, in this patient, it was delayed likely due to the increased pulmonary blood flow through fetal life and resultant changes in the pulmonary vasculature. In order to promote the pulmonary artery relaxation and drop in PVR, inhaled nitric oxide (iNO) and oxygen were administered for twenty‐four hours. With iNO and supplemental oxygen, clinical and echocardiographic evidence of RV hypertension improved quickly. Serial postnatal echocardiograms showed normalization in RV pressure within the first two weeks of life, followed by a gradual resolution of RV hypertrophy, and RV dysfunction over the first six months of life. This case illustrates that it is imperative to monitor patients with ductal constriction or closure throughout gestation to ensure delivery prior to the onset of right heart failure or hydrops fetalis. Postnatal management should focus on therapies to reduce PVR which will improve right heart hemodynamics and lead to less right‐to‐left PFO shunting and cyanosis. Fetuses that can escape this neonatal period without extreme prematurity, hydrops, or RV failure have good long‐term prognosis 9, 10.

In conclusion, this case highlights that PDA restriction or closure is a rare but a potentially severe complication of second‐trimester indomethacin use, despite prior reports indicating that indomethacin is a safe tocolytic at gestational age <32 weeks. In cases of premature PDA closure, serial echocardiograms are indicated to monitor the severity of the right heart dysfunction and for development of hydrops fetalis. Additionally, this case illustrates the characteristic fetal cardiac manifestations of premature PDA closure.

## Authorship

Jeanette I. Beaute authored the manuscript. In addition to editing the manuscript, Dr. Friedman diagnosed and treated the patient.

## Conflict of Interest

None declared.
